# A HTA of what? Reframing through including patient perspectives in health technology assessment processes

**DOI:** 10.1017/S0266462323000132

**Published:** 2023-05-18

**Authors:** Callum J. Gunn, Barbara J. Regeer, Teun Zuiderent-Jerak

**Affiliations:** Athena Institute, Faculty of Science, VU University Amsterdam, Amsterdam, The Netherlands

**Keywords:** Patient participation, patient knowledge, stakeholder engagement, integration, institutional policy

## Abstract

**Objectives:**

Despite increasing emphasis on the inclusion of patient input in health technology assessment (HTA) in Europe in particular, questions remain as to the integration of patient insight alongside other HTA inputs. This paper aims to explore how HTA processes, while ensuring the scientific quality of assessments, “make do” with patient knowledge elicited through patients’ involvement mechanisms.

**Methods:**

The qualitative study analyzed institutional HTA and patient involvement in four European country contexts. We combined documentary analysis with interviews with HTA professionals, patient organizations, and health technology industry representatives, complemented with observational findings made during a research stay at an HTA agency.

**Results:**

We present three vignettes which showcase how different parameters of assessment become reframed upon the positioning of patient knowledge alongside other forms of evidence and expertise. Each vignette explores patients’ involvement during an assessment of a different type of technology and at a different stage of the HTA process. First, cost-effectiveness considerations were reframed during an appraisal of a rare disease medicine based on patient and clinician input regarding its treatment pathway; in the second vignette reframing amounted to what counts as a meaningful outcome measure for a glucose monitoring device; in the third, evaluating pediatric transplantation services involved reframing an option’s appropriateness from a question of moral to one of legal acceptability.

**Conclusions:**

Making do with patient knowledge in HTA involves reframing of what is being assessed. Conceptualizing patients’ involvement in this way helps us to consider the inclusion of patient knowledge not as complementary to, but as something that can transform the assessment process.

## Introduction

### Evolution of HTA towards comprehensive evidence assessment

Recent discussions in HTA are focused on how assessment practices can become more attentive to the real-world impacts of health technologies. Commentaries suggest a range of directions in which HTA processes can evolve to maintain sustainable access to health care in response to changing healthcare evidence landscapes (1).

One strategy is the inclusion of different perspectives and the participation of different stakeholder groups – with patient input to HTA forming a key focus of current conversations. Some have emphasized that mechanisms of including patient perspectives throughout HTA lead to more relevant and robust evidence assessments (2) and contribute to better quality decision-making (3). The HTAi Interest Sub-Group for Patient/Citizen Involvement in HTA described multiple rationales for patient involvement as: legitimacy (of process); fairness (involving those impacted) and equity (understanding diverse needs); and relevance (robust, well informed) (4). Meanwhile, the role of patient (and public) involvement has become recognized internationally as an important element of HTA. The recent EU regulation on HTA solidified a commitment to including patient input as part of the expansion of collaboration in HTA. The regulation states that “external experts … includ[ing] patients affected by the disease” should provide input to assessments to “ensure that joint work is of the highest scientific quality” (p. 24) (5).

The participation of patient actors across different stages of assessment is seen as a major approach to the direct inclusion of patient perspectives in HTA. Various international initiatives have been involved in the development of mechanisms to establish patient involvement as part and parcel of HTA (6). In Europe specifically, following the HTA regulation, the European Network for HTA (EUnetHTA) seeks to further establish patient involvement in collaborative HTA activities and services. One working stream of EUnetHTA aims to develop guidance “for the interaction with and involvement of patient representatives” in its joint consultations and assessments (7). Because of the international commitment to the participation of patient actors in assessment processes, understanding how the barriers to patient involvement can be navigated in particular will be beneficial for the improvement of overall HTA practice.

Despite widespread acknowledgement of the importance of patients’ participation in the HTA process, scholars have long been concerned with the way that patient input – in its different forms – makes a difference to HTA (8;9). Despite being a part of HTA for some years, recent commentaries still speak of the guidance needed to embed patient involvement into institutional HTA practice (10).

An increasing collection of efforts and initiatives have sought to conceptualize what constitutes optimal patient involvement in HTA in different contexts (11;12). A few empirical studies have examined the factors affecting meaningful and impactful patient involvement in the HTA process (13;14). Often reported is the lack of time available for patient groups to contribute during assessment processes, with better (financial) support and resources considered key to meaningful participation (13;15). Bidonde et al. (14) highlight the more complex, epistemological issue regarding the challenges of integrating patient knowledge in the “institutional settings and intellectual traditions” that govern HTA practice. This typically manifests as difficulties in smoothly integrating patient input – often conceptualized in HTA as experiential, embodied, and articulated through personal stories (16) – alongside more established (e.g., clinical and economic) forms of evidence (3;10). The aforementioned multiple rationales for patient involvement ultimately mean assessment processes must often “make do” with the inputs provided by patient actors. As such, Bidonde et al. (p. 7) argue for a more pronounced “acknowledgement and recognition of other epistemic traditions, besides evidence-based medicine” to deal with the challenges of patient involvement in HTA.

Recent discourse implies that patient input is important for ensuring the scientific quality of HTA assessments (5). If this is the case, it has significant implications for thinking about how to give space to patient knowledge in HTA, where, generally speaking, appropriate evidence and knowledge are aggregated into an overall image of health technology in order to provide coherent advice on the best options for policy makers. To our knowledge, with a few exceptions (16), there are limited examples of studies that examine, in empirical detail, how HTA processes attempt to “make do” with patient knowledge in practice. Thus, there is scope to add to the debate about the (the impact of) patients’ involvement in HTA through exploring how room is made for patient knowledge to contribute to assessment outcomes alongside other forms of evidence and knowledge. The next section discusses the methods we employed to reach this aim.

## Methods

### Setting

This paper draws on data from a qualitative comparative study of four institutional HTA processes in Europe. The study combined document analysis with interviews with HTA professionals, patient organizations, and health technology industry representatives, in relation to national and collaborative HTA processes, as well as observations during a three-month research stay at an HTA agency in 2018.

### Collection

Interviews were held with ten HTA representatives (working at an HTA agency or as an academic researcher with experience conducting assessments), six patient organization representatives, and six respondents working in the health technology industry. Interview respondents were based in four countries in western Europe: Ireland, the Netherlands, Norway, and Scotland. Country contexts were selected purposively based on their history with HTA assessments where patient input played a notable role.

Interviews explored the HTA process in each national context and discussed the challenges and opportunities to meaningful patients’ involvement. Relevant documents analyzed included final HTA reports and advice, guidelines for HTA practice including patient involvement, patient evidence submissions and templates, and other relevant policy documents or press releases related to HTA in the selected country contexts.

Access to HTA and some patient organization representative respondents was provided through the first author’s research stay at an HTA agency in 2018. Collection and initial sense-making of the data were complemented through observational findings during this time. This included discussions with staff at the hosting institute and non-participant observation of the internal institute meetings (*n* = 6), with field notes made throughout.

All interviews were recorded and transcribed verbatim. All interviewees gave oral and written consent to use of interview data prior to their participation. Any direct quotations of respondents are anonymized to ensure confidentiality. A formal waiver for ethical approval was obtained through the Faculty of Sciences ethics self-assessment tool at Vrije Universiteit Amsterdam.

### Analysis

*Documentary analysis.* After an initial round of open coding to organize the data, axial coding developed categories and their relations (17). This informed the analysis of initial interviews that involved a separate round of open coding to explore the effects of patient involvement on HTA, and factors influencing effective patient involvement processes. Observational findings were compared with the concepts emerging from initial interviews and documentary analysis, with their relevance checked with practitioners in the field and searches for disconfirming evidence (18). Analysis was theoretically guided by a research-in-practice perspective (19;20), which emphasizes the specific working arrangements and techniques of integrative scientific work including the cooperation between different perspectives. Coding was facilitated through Atlas.ti.

During coding rounds, we sought to understand how different contextual aspects could play a role in the integration of different types of knowledge in HTA. We considered reported contextual variants when comparing different assessments (21), including type of technologies being assessed, and the timing of patients’ involvement in the HTA process. Through asking these kinds of questions during this stage, we found three specific exemplary situations that provide insights into how patients’ involvement and knowledge integration happen in HTA in different ways. We present these as separate “vignettes”: we highlight key moments of patients’ involvement in the HTA process. These specific instances of patient involvement in HTA were chosen as most illustrative of the themes that emerged during the coding of material.

The first vignette is an assessment of an orphan medicine for a rare blood disorder affecting kidney functioning. Here, the key moment of patient involvement happened late in the process, during the appraisal phase following the assessment of available evidence. The second is a continuous glucose monitoring system for type 1 and 2 diabetes mellitus patients, and we focus on the involvement of patients that took place at the start of a relative effectiveness assessment. The third is an assessment of options for emergency pediatric organ transplantation services. Here, the notable moment of patients’ involvement we analyze happened as the evidence assessment was ongoing.

The outcomes of the vignettes were then compared with the initial categories to refine the interpretations. We further ensured validity through our engagement with the HTA field through checking of initial empirical analysis with practitioners. Contributing participants of the final vignettes were contacted to check the accuracy and relevance final accounts for accuracy and relevance, with no additions or changes being requested to the final manuscript.

In the following section, we present three different vignettes of patient involvement: here it is important to note that using the vignettes is not intended as a generally representative picture of the outcomes of patient involvement in HTA, but rather their function is about finding and presenting examples that provide specific insight while teaching us something about a more general issue at stake (22). We utilize vignettes thus because (i) the context of the involvement should not be sidestepped in its analysis – including the specific technology under assessment, the general stage of the HTA, and the processes by which patient perspectives were collected and appraised in the assessment – and therefore (ii) this shows the variant ways that the presence of different knowledge holds implications for the way that judgments are made about health technologies during the HTA process. While the vignette descriptions provide useful background, we do not intend to sketch a comprehensive account of the assessment, or the inclusion of patients’ perspectives therein.

## Results

The vignettes offer a spectrum of different “types” of technology and patients’ involvement. Each are examples where the final recommendation was to approve the reimbursement of the technology in some form, with the outcomes of each assessment published as publicly available HTA reports.

### Vignette 1: orphan medicine for rare blood disorders (eculizumab)

The assessment of a treatment for rare, life-threatening genetic diseases that cause blood disorders was conducted in the Netherlands in 2016 by Zorginstituut Nederland (ZINL), with the processes of this appraisal being analyzed elsewhere (23). We zoom in on the appraisal phase, where a committee formed of experts from different domains (the appraisal committee), including patient and clinical representatives, considers the final technology assessment report in formulating its recommendations for reimbursement of the technology. This deliberative phase explicitly aims to weigh up arguments arising from the review of scientific evidence with ethical and societal arguments and concerns. Patient groups sit as experts on the deliberative committee (24).

In the context of the assessment, patient and clinical representatives felt that the medicine’s treatment pathway could be different for many patients, who would only require eculizumab treatment in emergency circumstances rather than as a regular, life-long treatment regimen. Together, these actors developed an alternative proposal regarding the medicine’s treatment guideline – specifically its administration protocol with different start and stop criteria – and submitted this to the appraisal committee. This proposed alternative protocol formed the “primary focal point” (23) of the deliberations among the committee involved in formulating the advice based on the earlier evidence assessment. The incremental cost-effectiveness ratio (ICER) was calculated based on the European Medicines Agency (EMA) -approved treatment guideline that, at that time, stated that the treatment should be provided once every 2 weeks for the rest of the patient’s life (25). While the original calculated ICER was considered highly unfavorable, meaning it would be unlikely that the reimbursement of the technology on public insurance would be recommended, the appraisal committee decided to conditionally recommend reimbursement on the basis of the alternative treatment protocol.

Referring to this example, a patient organization representative stated:

“Here the appraisal discussion changed because the patient group and physicians operated together […] these are examples of where early involvement of stakeholders could have had a totally different impact on the [technical assessment] process.”

Although it may have led to the same outcome, this response implies that the involvement of patients in the effectiveness assessment would have impacted the initial evaluation and ICER calculations *before* these adjustments that were deemed necessary.

The advice changed here because the consideration of what the therapy consists of was redefined*.* The therapy’s effectiveness was *reframed* through an alternative administration strategy, leading to a more favorable ICER and thus an improved value for money. So, what happens when patients’ knowledge is brought in earlier in the HTA process, during the effectiveness assessment for instance? This brings us to the next vignettes.

### Vignette 2: continuous glucose monitoring system

A subcutaneous, continuous glucose monitoring system technology (CGM), for type 1 and 2 diabetes mellitus patients, was assessed in Norway (Norwegian Institute of Public Health [Folkehelseinstitutttet] – NIPHNO) 1 year prior to Scotland (Scottish Health Technologies Group – SHTG). Both HTA agencies performed clinical and cost-effectiveness assessments of the technology. Both assessments compared the CGM against conventional self-monitoring of blood glucose.

In the Norwegian context, patient perspectives had been articulated in scoping meetings held at the beginning of the assessment project. These meetings have an aim to develop and improve the formulation of assessment questions. The standard approach scoping the questions of effectiveness assessments uses the PICO (Population, Intervention, Comparator, and Outcomes) framework. In this case, drafting the PICO criteria was executed in collaboration with clinical experts and a national diabetes patient organization. Since using the CGM could supposedly reduce/remove the performance of multiple daily injections in monitoring patients’ blood glucose levels, representatives highlighted the effect on the pain experienced by patients as an important outcome for understanding the technology’s impact. This informed the assessment’s evidence review. However, there were limited reports measuring pain as discrete effect outcome, although some studies had recorded pain as part of adverse events of using the CGM.

“[the problem] wasn’t actually the report itself, it was more the way of doing the HTA and the evidence available…that has something to do with our tradition, we used to be a kind of quantitative evidence institute, looking at the effective interventions, and to look at effective interventions we have these randomized controlled trials as being the gold-standard.” (HTA researcher)

This remark identifies the problem as being the way that evidence credibility standards are imposed in HTA, which meant little could be confirmed about the issue of pain at the time.

“Pain [from using the technology] was seen from the patient input as a really important outcome. But we didn’t find any evidence to address it, not really…”. (HTA researcher)

As this response suggests, the final assessment report included acknowledgement of these issues that could not be fully addressed due to a lack of available information.

That being said, the “suggested research priorities” section of the final Norwegian report recommended that evidence generation activities in future should measure pain (and its effect on treatment adherence) as part of the impact of the CGM compared to conventional glucose monitoring (26). Similarly, the final report in Scotland (p. 19) encouraged future research to make use of data collected within electronic patient records to “inform future assessments” (27) of the CGM and other similar technologies. Overall, these recommendations, somewhat influenced by patient input to these particular assessments in 2017/2018, are focused on re-defining the definition of a meaningful outcome measure for future evaluations of diabetes-related health technologies.

### Vignette 3: pediatric organ transplantation service

In 2017, the Health Information and Quality Authority (HIQA), the Irish HTA agency, was requested to conduct a “rapid HTA evaluating the treatment and transport options” for emergency pediatric heart and liver transplant patients. At the time of the assessment, children urgently requiring a transplant were transferred from Ireland to the surgical location in the UK via air ambulance. Capacity constraints meant that current transfer service arrangements could no longer be available. The scope of the assessment was to “set out the alternative approaches for providing efficient and sustainable treatment or transport of these patients, and provide a high-level assessment of the clinical, economic and organizational consequences of the alternative approaches for the treatment or transport of these patients” (28) (p. 4).

The assessment was described as unusual since it explicitly omitted a cost-effectiveness analysis due to the complexity of the service being evaluated, with all possible options considered as having a “substantial budget impact.”

Patient representatives sat within an expert advisory committee – this group consisted of experts from different disciplines and organizations set up to guide the assessment process and contribute to the overall HTA recommendations. Among other things, the advisory group was expected to “provide advice to [HTA agency] regarding the scope of the analysis [and] support the evaluation team during the assessment process by providing expert opinion” (28) (p. 5).

Interviewees noted that the perspective of patients, through personal experience, added rich detail to the interpretation of different situation, and thus the consideration of possible options for transport among the advisory group.

A patient organization representative told us about a specific moment in an expert advisory group meeting during the assessment. The group (which included the interviewee below as well as other patient representatives) was discussing the implications of different transport options with the HTA evaluation team. One proposed option involved the parent or guardian driving the patient to the air ambulance site behind a police escort. A representative raised an example of another parent transporting their child to the emergency transfer site from their home via this option. The representative conveyed the experience of the parent to the expert group, that involved driving at excessive speeds during the middle of the night. The parent reflected on their experience as extremely distressing. While the group responded to the significance of the story, there were concerns about the extent to which the severity of this specific experience could be extrapolated to all patient/caregiver situations. However, the experience was then interpreted by another committee member, who raised a legal consideration in relation to the parent’s story. The member representing an emergency service organisation contended that a state agency cannot ask a civilian to break the law, which is what had happened. According to our respondent, this intervention ‘changed the discussion’.

As a legal matter, the acceptability of the option had been reframed as something more clearly problematic. In the context of HTA, this reframing had significant consequences for the interpretation of the option’s acceptability. Initially a question of whether the option could be deemed morally permissable, this became reframed as a question of legal acceptability as a result of the interaction between different stakeholder knowledge and expertise.

The three vignettes presented in this paper can be summarized as exemplars for making sense of the main challenge explored in this paper: “Making do” with patient knowledge in HTA processes fundamentally involves *reframing* – a reframing of what is being assessed through the positioning of different forms of knowledge and expertise in HTA. In the assessment of the orphan medicine, patient knowledge helped to define the criteria used for administering the drug, or what “the therapy” consists of. This revised interpretation of the technology reframed the consideration of its value for money. In the diabetes sensor assessment, patient input reframed what counts as effectiveness. While the assessments at that time did not have adequate evidence to support patients’ preferred outcome measurement, the recommendations sought to utilize patient knowledge in an “upstream” manner – to adapt the criteria for determining effectiveness in later evidence generation practices. In the final vignette, the appropriateness of one transport option being considered was reframed from a question of moral to one of legal acceptability. The consequence of the reframing was that the patient experience being articulated, despite being a singular example, could be considered as a generic matter of legal acceptability of the option being assessed.

The temporal aspect of each vignette is important for understanding how reframing relates to ensuring high scientific quality in HTA. In the first vignette, patients’ involvement happens after the assessment process – during the weighing up of “the evidence” with other societal and ethical arguments. In the second, involvement reframes what later evidence generation (and thus future HTA evaluations) should look for in terms of outcome measures – in this way it happens prior to the scientific process. In the third, involvement happens during the scientific process as the evaluation team analyzes and interprets the evidence base together with patient and other experts in the advisory group. Importantly, including different perspectives serves to modify the evidence base and the way it is analyzed in the HTA process.

## Discussion and conclusion

The added value of including patient knowledge in HTA is well recognized. If patient input is indeed necessary to ensuring the “highest scientific quality” in HTA (5), it seems critical at this point to consider what it would take for HTA to handle the challenges of integrating different forms of evidence and knowledge.

Scholars and practitioners involved in HTA are acutely aware of the tensions raised by including and assessing patient input with technology assessments (3). A variety of efforts have been developed in approaching the methodological concerns relating to the integration and utilization of patient inputs in a scientifically robust way in HTA. Many have argued for the generation of more “robust” forms of patient input for HTA (3;29), as sources of so-called ‘real-world evidence’, in particular through the development of capacities (e.g., within patient organizations) to generate a more patient-centered evidence base. Other work has focused on using patient involvement to scope assessment questions based on PICO criteria such as identifying patient-relevant outcomes (30). However, with the complexities associated with patient stories and narratives, patients’ involvement tends to be framed as supplementary or complementary to the HTA process, as providing context and reassurance for, rather than directly impactful to, HTA recommendations and decision-making processes (31).

Meanwhile, calls are being voiced for a more profound integration of the empirical and normative elements of HTAs (32). These perspectives argue that scientific assessments of health technology need to deal with the complex interlinkages of technical, social, and ethical questions raised by health innovations (33). Our findings provide empirical support to these arguments and may suggest how patient involvement can mobilize an integrative approach in institutional HTA practice (10).

Our findings highlight how, through patients’ involvement, the positioning of different forms of evidence and expertise alongside one another leads to a reframing of what is being assessed in HTA. Reframing different assessment parameters – what the technology consists of, its effects, and its acceptability, as shown in the vignettes presented – leads to the adaptation of the evidence base and how it is interpreted throughout the HTA process. In this way, we can observe how the interaction of different stakeholder perspectives and knowledge within HTA blurs the boundaries between empirical (i.e., technical/scientific) and normative (i.e., social/ethical) analysis and judgment of health technologies and their added value.

While we recognize the limitations of drawing generic conclusions from specific vignettes, conceptualizing patients’ involvement in this way helps us to consider how the inclusion of patient knowledge can transform the HTA process rather than being complementary to it. This refers to transformation of relevant evidence bases and how HTA goes about analyzing them. Making room for different inputs means the *problem space is shared* (34) – between different actors, knowledges, and perspectives that contribute to collectively making sense of health technology’s value in relation to the evidence base (16). Such a configuration of stakeholders’ engagement in HTA would embody a more adaptive and flexible approach to the assessment of health technology (33), but the epistemic and institutional setting of HTA needs to be taken seriously in any discussion about transforming assessment processes (14).

To improve conceptualizing of transformative reframing, the role and status of other stakeholder knowledge, alongside that of patient actors, will be important to investigate further. This study has focused on patients’ knowledge as the key agent in reframing process, but exactly how patient knowledge is legitimized and mobilized by other stakeholders’ input and actions (for instance, *how* clinician and patient actors produced an alternative protocol in vignette 1, which remained ambiguous in our dataset) remain open for examination by further in-depth qualitative research of HTA processes. This is where consideration of other contextual factors will be important. For instance, in these vignettes, we assume that patient groups were relatively well resourced, information-wise, to contribute to the HTA process. Patient groups’ (access to) resources will be different in different contexts of HTA and patient involvement. Patient involvement does not equate to reframing and/or impact, and the reasons behind examples where patient involvement makes no material difference to assessment processes are just as interesting to examine to improve our conceptualization of reframing through including patient knowledge in HTA.

## Conclusion

Patients’ understandings of technologies in practice, in “real-world” settings, connect with clinical, economic, and other forms of knowledge and expertise in the formation of HTA’s analyses of health technology. The implications of connecting different evidence and expertise – making do with patient knowledge – are that, to different extents, “assessments” of health technologies become challenged, revised, and reframed. If including patients’ input is important for ensuring scientific quality in HTA, without such an understanding of reframing what is being assessed, patient involvement will tend to be underestimated by remaining complementary.
